# Assessment of the incremental prognostic value from the modified frailty index-5 in complete traumatic cervical spinal cord injury

**DOI:** 10.1038/s41598-023-34708-5

**Published:** 2023-05-10

**Authors:** Husain Shakil, Blessing N. R. Jaja, Peng F. Zhang, Rachael H. Jaffe, Armaan K. Malhotra, Erin M. Harrington, Duminda N. Wijeysundera, Jefferson R. Wilson, Christopher D. Witiw

**Affiliations:** 1 Division of Neurosurgery, Department of Surgery, University of Toronto, Toronto, M5T1P5 Canada; 2 St. Michael’s Hospital, Li Ka Shing Knowledge Institute, Toronto, M5B1T8 Canada; 3 Division of Neurosurgery, Department of Surgery, St. Michael’s Hospital, Toronto, M5B1W8 Canada; 4 Institute of Health Policy Management and Evaluation, University of Toronto, Toronto, M5T1P8 Canada; 5 Department of Anesthesia, St. Michael’s Hospital, Toronto, M5B1W8 Canada; 6 Department of Anesthesiology and Pain Medicine, University of Toronto, Toronto, M5T1P8 Canada

**Keywords:** Geriatrics, Trauma, Patient education, Prognosis, Outcomes research, Risk factors

## Abstract

Frailty, as measured by the modified frailty index-5 (mFI-5), and older age are associated with increased mortality in the setting of spinal cord injury (SCI). However, there is limited evidence demonstrating an incremental prognostic value derived from patient mFI-5. We conducted a retrospective cohort study to evaluate in-hospital mortality among adult complete cervical SCI patients at participating centers of the Trauma Quality Improvement Program from 2010 to 2018. Logistic regression was used to model in-hospital mortality, and the area under the receiver operating characteristic curve (AUROC) of regression models with age, mFI-5, or age with mFI-5 was used to compare the prognostic value of each model. 4733 patients were eligible. We found that both age (80 y versus 60 y: OR 3.59 95% CI [2.82 4.56], *P* < 0.001) and mFI-5 (score ≥ 2 versus < 2: OR 1.53 95% CI [1.19 1.97], *P* < 0.001) had statistically significant associations with in-hospital mortality. There was no significant difference in the AUROC of a model including age and mFI-5 when compared to a model including age without mFI-5 (95% CI Δ AUROC [− 8.72 × 10^–4^ 0.82], *P* = 0.199). Both models were superior to a model including mFI-5 without age (95% CI Δ AUROC [0.06 0.09], *P* < 0.001). Our findings suggest that mFI-5 provides minimal incremental prognostic value over age with respect to in-hospital mortality for patients complete cervical SCI.

## Introduction

Complete traumatic cervical spinal cord injury (SCI) confers significant in-hospital mortality risk to patients. Mortality after such an injury has been estimated as high as 26%^1–3^. Several studies have sought to identify factors contributing to this high rate, and unsurprisingly, patient age often emerges as a strong predictor of mortality^4–9^. However, in recent years there has been growing interest in the concept of clinical frailty as a counterpart to patient age in predicting patient morbidity and mortality^10^.

Clinical frailty refers to a state of decreased physiologic reserve and vulnerability to stressors due to a decline in the normal functioning of multiple organ systems^11^. Numerous scales have been described to quantify the degree of frailty in a patient^12–14^. In the setting of trauma, the 5-item modified frailty index (mFI-5) has been described as a facile tool to determine a given patient’s frailty^13^. The mFI-5 is calculated based on the presence of diabetes, hypertension, congestive heart failure, chronic obstructive pulmonary disease, and dependent functional status. In cases of traumatic SCI, the mFI-5 has been shown to be a predictor of in-hospital mortality^5^.

A state of high frailty is generally associated with older age, and vice versa, but there are always exceptions to the rule. There are invariably cases of high functioning elderly patients with minimal chronic disease, and by contrast, middle aged individuals with poor nutrition, mobility, and numerous comorbidities. As such, there is not necessarily a one-to-one correlation between a given patient's age and frailty. In the setting of degenerative cervical myelopathy, frailty provides incremental prognostic value over evaluation of a patient’s age^32^. However, to our knowledge, no study has compared the role of age and frailty in evaluating in-hospital mortality in the context of SCI. In this study we aim to address this knowledge gap, by using a large multi-center database to assess whether mFI-5 provides incremental prognostic value as a predictor of in-hospital mortality.

## Methods

### Data source

All data in this study were derived from the 2010–2018 American College of Surgeons (ACS) Trauma Quality Improvement Program (TQIP)^15,16^. More than 450 ACS- and state-verified level I and II trauma centers across North America contribute to TQIP. It includes all patients from verified centers with at least one severe injury (Abbreviated Injury Scale [AIS] ≥ 3 in at least one body region). Data reliability and quality are maintained through training of data abstractors and inter-rater reliability audits of contributing centers.

### Research ethics board approval

This study number 20-247 was approved by the Unity Health Toronto Research Ethics Board (Toronto, Ontario, Canada) in January of 2021. Study procedures were followed in accordance with the ethical standards of the institutional committee on human experimentation and with the Helsinki Declaration of 1975. This study used only de-identified retrospective patient data, and individual participant informed consent was waived by the Unity Health Toronto Research Ethics Board.

### Study eligibility

Adult patients (≥ 16 years) with a diagnosis of acute complete (ASIA A) traumatic cervical SCI due to blunt trauma that were treated at level I or II trauma centers were included based on AIS codes (Supplementary Table S1). The International Classification of Diseases 9th and 10th revision Procedure Classification System (ICD-9-PCS and ICD-10-PCS) codes were used to identify procedure codes for decompression and fusion (see Supplementary Table S2). Patients with missing data on whether they underwent spinal surgery were excluded. In addition, patients with missing data on in-hospital mortality were also excluded as this was our primary outcome of interest. Finally, patients with any AIS body score of 6 were also excluded, as these are considered non-survivable injuries^17^.

### Statistical analyses

All statistical analyses were performed using R version 4.2.1 with an a priori specified significance level of *P* = 0.05 (two-tailed). Descriptive statistics were reported as mean and standard deviation (SD) for continuous variables and count and percentage for categorical variables.

#### Patient, injury, treatment, and hospital characteristics

Several patient and hospital covariates were selected from the TQIP database according to their clinical relevance as defined by prior studies^18^. Patient demographic data included age, mFI-5, sex, ethnicity, and insurance type. Age was considered a continuous variable. The mFI-5 is a frailty index that has been used in trauma and is scored with one point given based on the presence of each of the following: diabetes, hypertension, congestive heart failure, chronic obstructive pulmonary disease, and dependent functional status^19^. We dichotomized patients into categories of low frailty (mFI < 2) and high frailty (mFI ≥ 2). This type of dichotomy in the mFI-5 has been found to be relevant in prior studies^5^. Sex was dichotomized into male and female, and ethnicity was grouped into categories of African American, Caucasian, and other. Insurance was categorized as private, public, and other. Data on the characteristics of the injury were also collected. This included mechanism of injury, presenting Glasgow Coma Scale (GCS), presence of hypotension (defined as emergency department blood pressure < 90 mmHg), and year of injury. The patient’s GCS was categorized as GCS15, GCS13-14, GCS 9-12, and GCS 3-8, consistent with categories corresponding to severity of traumatic brain injury^20^. Mechanisms of injury were categorized as motor vehicle traumas, falls, and other. The primary treatment characteristic extracted from TQIP was whether the patient underwent a spinal operation. We used ICD-9 and -10-PCS codes as described above to identify patients who underwent a spinal operation. Surgery was therefore classified as a binary variable. Hospital characteristics including the ACS verification level, teaching status, and hospital size were also extracted from TQIP. Hospital teaching status was categorized as university hospital, community hospital, and non-teaching hospital. Hospital size was categorized as < 200 beds, 200-400 beds, and > 400 beds. Variable categories were chosen largely based on prior studies^18^.

#### Outcomes

The primary outcome was in-hospital mortality during the trauma admission. We computed counts and proportions of mortality across our age range and frailty categories. We computed a heat map of unadjusted mortality rates using the *lattice* package, and by binning patients into age decades (i.e., 16–20 years, 21–29 years, 30–39 years, 40–49 years, 50–59 years, 60–69 years, 70–79 years, ≥ 80 years)^21^. The unadjusted mortality rate was computed as the ratio of in-hospital deaths by the total number of study patients within each age decade and mFI-category.

#### Regression modelling

The *rms* package was used to perform multivariable logistic regression to model in-hospital mortality^22^. Patient age, mFI-5, sex, ethnicity, insurance type, mechanism of injury, presenting GCS, presence of shock, whether they underwent surgery, hospital ACS verification level, teaching status, hospital size, and year of injury were used as covariates for adjustment. Age was modelled as a non-linear function using restricted cubic splines with 4 knots located at each quartile for age. Age was chosen to be modelled as a non-linear variable in reference to regression modelling methodology described by Harrell^22^. Specifically, we noted (1) a significant association between the non-linear term for age and the log-odds of mortality from an analysis of variance of predictor terms (see Supplementary Table S3); (2) a significant result in the likelihood ratio test comparing a model with age modelled as a linear covariate compared to a model with age modelled as a non-linear covariate (see Supplementary Table S4); and (3) a non-linear relationship noted on inspection of a plot of the adjusted log-odds of in-hospital mortality as a function of age (see Supplementary Figure S5).

We initially included an interaction term for mFI-5 and age by following the method described by Wilson et al. and Baron and Kenny^23,24^. However, in reference to regression modelling methodology described by Harrell^22^, we excluded the interaction term in the final model after (1) failing to observe a significant effect from interaction terms in the fitted model (see Supplementary Table S6); and (2) failing to find a significant difference in the log likelihood of a model with and without the interaction term (see Supplementary Table S4).

Fitted models were assessed by calculating the odds ratio for each covariate in the model. For age, the odds ratio associated with an age change of 60–80 years is reported. This range was chosen through inspection of the fitted probability curve for in-hospital mortality as a function of age. We chose to report the odds ratio with the 20-year interval associated with the largest magnitude of change in mortality probability. The *p*-values associated with each covariate/predictor was computed using an analysis of variance.

We created a ranked Forest plot to compare odds ratios associated with different predictors in the full model. We ranked predictors after grouping by covariate type: (1) Patient characteristics: age, sex, ethnicity, insurance type; (2) Injury/Presentation characteristics: mechanism of injury, GCS, presence of hypotension; (3) Treatment characteristics: whether a patient underwent surgery; and (4) Hospital characteristics: bed size, teaching status, and ACS verification level. Within the Forest plot, we plotted the odds ratio associated with an age change from 60-80 years, consistent with the range reported for the full model.

#### Assessing incremental prognostic value from mFI-5

Three separate logistic regression models were compared to assess the incremental prognostic value of mFI-5. We first composed a full model including age and mFI-5 as covariates along with 11 base covariates: sex, ethnicity, insurance type, mechanism of injury, presenting GCS, presence of shock, whether they underwent surgery, hospital ACS verification level, teaching status, hospital size, and year of injury. We then created a nested model including only age and the 11 base covariates as independent predictors in the model. Lastly, we created a second nested model including only mFI-5 and the 11 base covariates as independent predictors. To assess incremental prognostic value of mFI-5 we compared the three models with respect to common regression model discrimination and reliability indices described by Harrell^22^. We compared the log-likelihood of each model using the likelihood ratio test. We used the *pROC* package to generate a receiver operating characteristic (ROC) curve for each model. The area under the ROC curve (AUROC) of each model was compared using Eq. (1).

1 $${\triangle\text{AUROC}} = {\text{AUROC}}_{{{\text{Full}}\,{\text{model}}}} - {\text{ AUROC}}_{{{\text{Nested}}\,{\text{model}}\,{\text{i}}}}$$

The 95% confidence interval and *p*-value comparing each AUROC was computed using DeLong’s method^25^, and a stratified bootstrap with 2000 iterations. The Somer’s D_xy_ rank correlation index, and Brier’s score was computed using a bootstrap technique with 500 iterations^22^. Lastly, we computed the fit of each model using Akaike’s Information Criterion (AIC)^26^.

We performed sensitivity analyses by completing an equivalent assessment of the incremental prognostic value from mFI-5 among two patient subgroups. Subgroup 1 was formed by restricting the cohort to patients with age above 60 years. Subgroup 2 was formed by restricting the cohort to patients with age above 60 years, and those who underwent surgery.

## Results

### Overview of cohort

**Figure 1:**
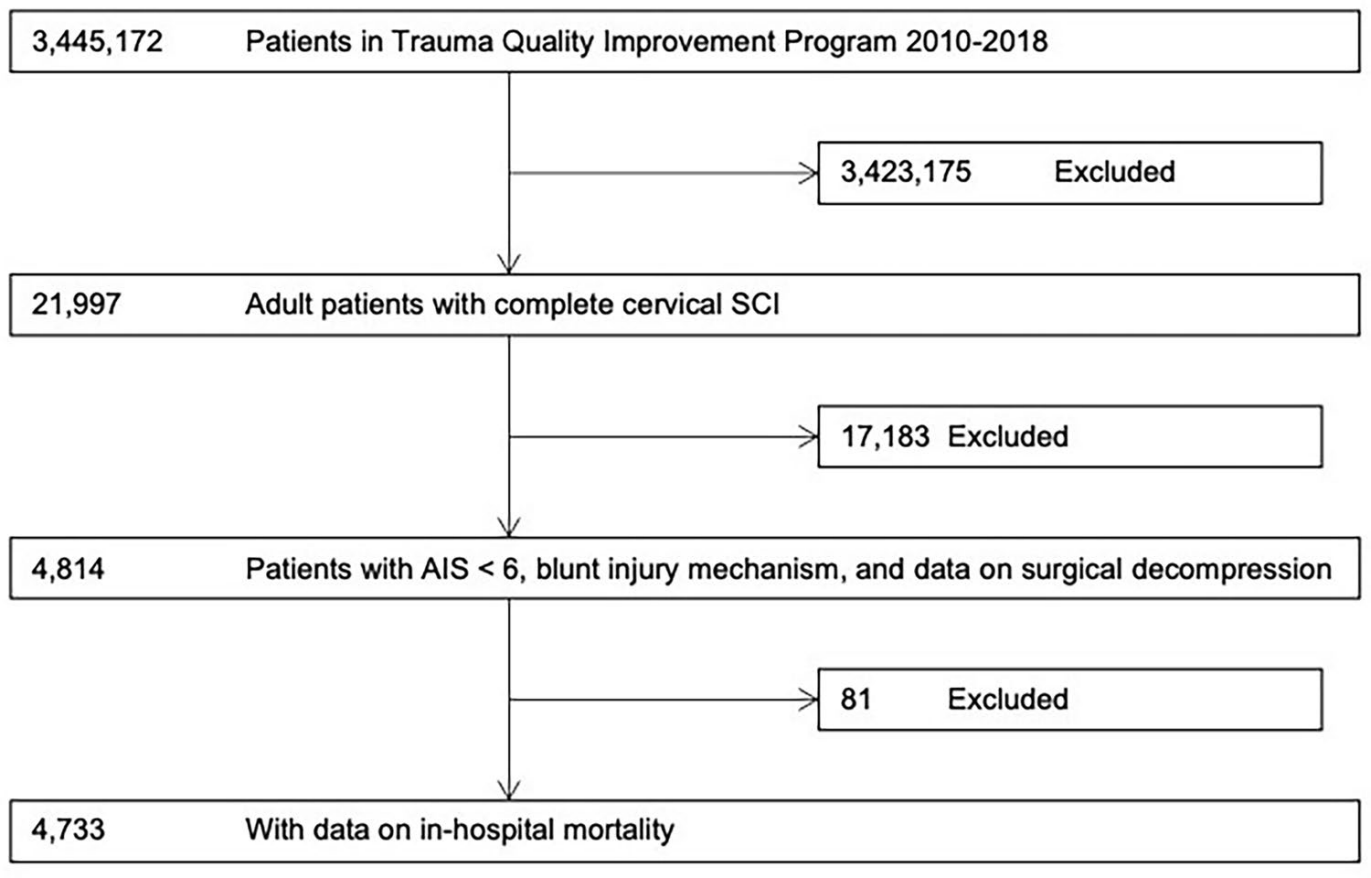
Flowchart of patient eligibility and enrollment.

Within the 2010–2018 TQIP database, we identified 4733 patients that sustained complete cervical SCI due to blunt trauma with survivable injuries with data on in-hospital mortality (Figure 1). Baseline characteristics of the cohort are summarized in Table 1. Mean age was 49.05 years (20.20 years SD); with 3803 (80.37%) males. Within the cohort 3843 (81.20%) patients underwent a decompression and/or a fusion procedure. With respect to frailty categories, 4052 (85.67%) patients with lower frailty (mFI-5 < 2); and 678 (14.33%) with high frailty (mFI-5 ≥ 2).

**Table 1:** Baseline characteristics of study cohort.

	Total Cohort—N = 4733	In-hospital mortality (Yes)—N = 751	In-hospital mortality (No)—N = 3982
Patient characteristics			
Age (years)-mean ± SD	49.05 ± 20.20	63.72 ± 18.56	46.34 ± 19.32
Modified frailty index-5 (mFI) category-n (%)			
Low Frailty (mFI < 2)	4052 (85.67)	554 (73.77)	3498 (87.91)
High Frailty (mFI ≥ 2)	678 (14.33)	197 (26.23)	481 (12.09)
Sex-n (%)			
Female	929 (19.63)	138 (18.38)	791 (19.87)
Male	3803 (80.37)	613 (81.62)	3190 (80.13)
Ethnicity– n (%)			
African American	992 (21.25)	111 (15.14)	881 (22.38)
Caucasian	3038 (65.07)	546 (74.49)	2492 (63.31)
Other	639 (13.69)	76 (10.37)	563 (14.3)
Insurance– n (%)			
Government	2016 (43.50)	406 (55.39)	1610 (41.27)
Private	2125 (45.86)	242 (33.02)	1883 (48.27)
Other	493 (10.64)	85 (11.6)	408 (10.46)
Injury/presentation characteristics			
Mechanism of injury– n (%)			
Fall	2205 (46.80)	388 (52.15)	1817 (45.79)
Motor vehicle trauma	1790 (37.99)	273 (36.69)	1517 (38.23)
Other	717 (15.22)	83 (11.16)	634 (15.98)
GCS– n (%)			
15	2865 (61.18)	350 (47.55)	2515 (63.72)
13–14	654 (13.97)	98 (13.32)	556 (14.09)
9–12	424 (9.05)	68 (9.24)	356 (9.02)
3–8	740 (15.80)	220 (29.89)	520 (13.17)
Hypotension in ED (SBP < 90)-n (%)	789 (16.90)	162 (21.77)	627 (15.98)
Year-n (%)			
2010	204 (4.31)	19 (2.53)	185 (4.65)
2011	270 (5.70)	29 (3.86)	241 (6.05)
2012	276 (5.83)	30 (3.99)	246 (6.18)
2013	288 (6.08)	25 (3.33)	263 (6.6)
2014	333 (7.04)	33 (4.39)	300 (7.53)
2015	391 (8.26)	41 (5.46)	350 (8.79)
2016	453 (9.57)	49 (6.52)	404 (10.15)
2017	1311 (27.70)	270 (35.95)	1041 (26.14)
2018	1207 (25.50)	255 (33.95)	952 (23.91)
Treatment characteristics			
Surgery-n (%)	3843 (81.20)	446 (59.39)	3397 (85.31)
Hospital characteristics			
Level I trauma center-n (%)	2927 (71.25)	416 (67.86)	2511 (71.85)
Number of beds-n (%)			
> 400	906 (19.15)	180 (23.97)	726 (18.24)
200–400	1576 (33.31)	234 (31.16)	1342 (33.71)
≤ 200	2250 (47.55)	337 (44.87)	1913 (48.05)
Teaching status-n (%)			
Community	155 (32.95)	253 (33.87)	1301 (32.78)
Non-teaching	471 (9.99)	90 (12.05)	381 (9.6)
University	2691 (57.06)	404 (54.08)	2287 (57.62)

SD, standard deviation; n, categorical variable count; GCS, Glasgow Coma Scale; ED, emergency department; SBP, systolic blood pressure.

### Frailty and age are associated with increased in-hospital mortality

**Figure 2:**
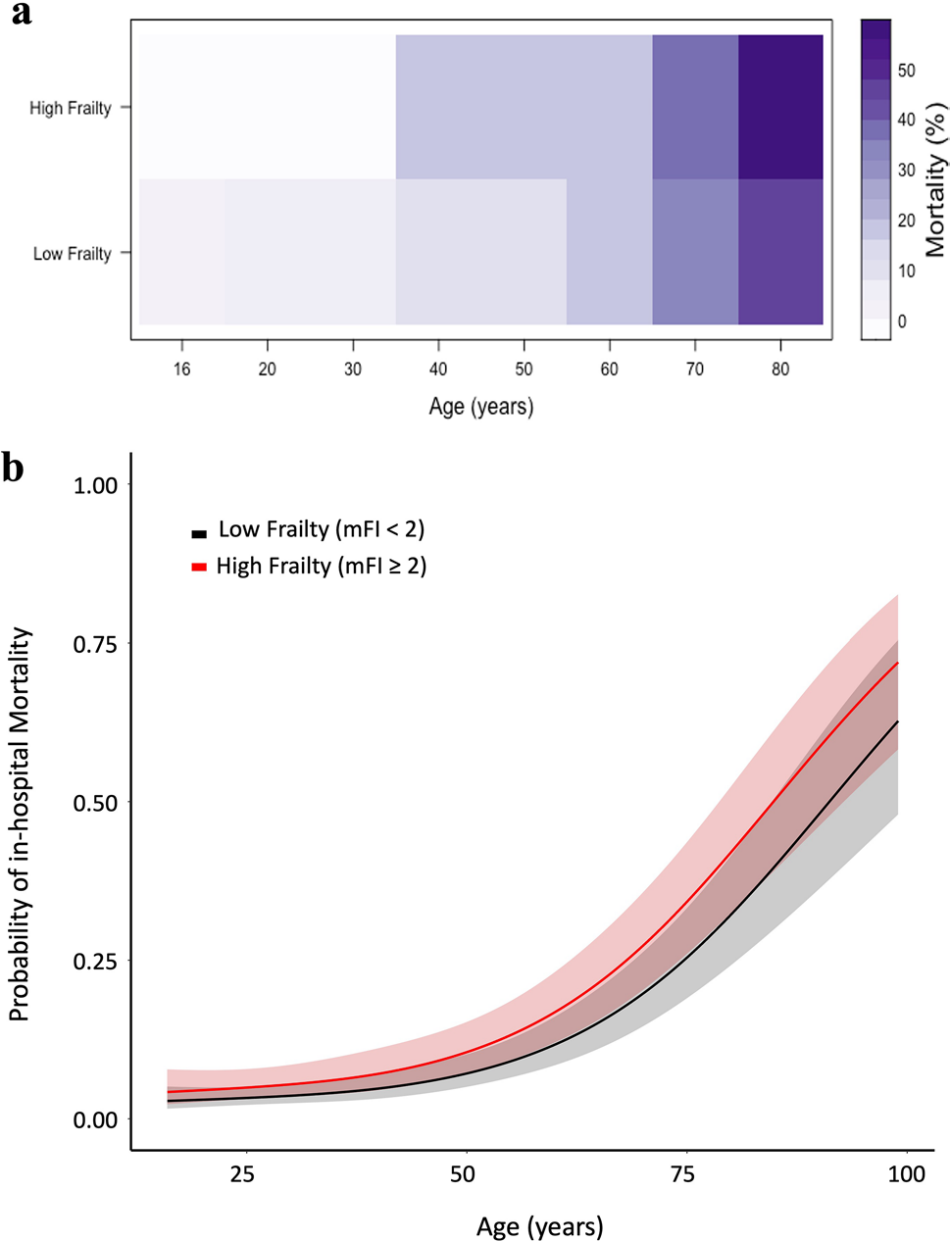
Mortality Increases Proportionate to Frailty and Age. (**a**) Heatmap of mortality proportions relative to age and Modified Frailty Index-5 (mFI) category. Low frailty mFI < 2; High frailty mFI ≥ 2. (**b**) Results from the full logistic regression model demonstrating the probability of an in-hospital mortality as a function of age and stratified by mFI-5 categories. Plots are adjusted to: Male sex, Caucasian ethnicity, private insurance, absence of hypotension, fall as mechanism of injury, GCS 15, hospital bed size ≤ 200, Level 1 American College of Surgeon verification level hospitals, university hospitals, injury year 2017, and patients undergoing surgery.

**Figure 3:**
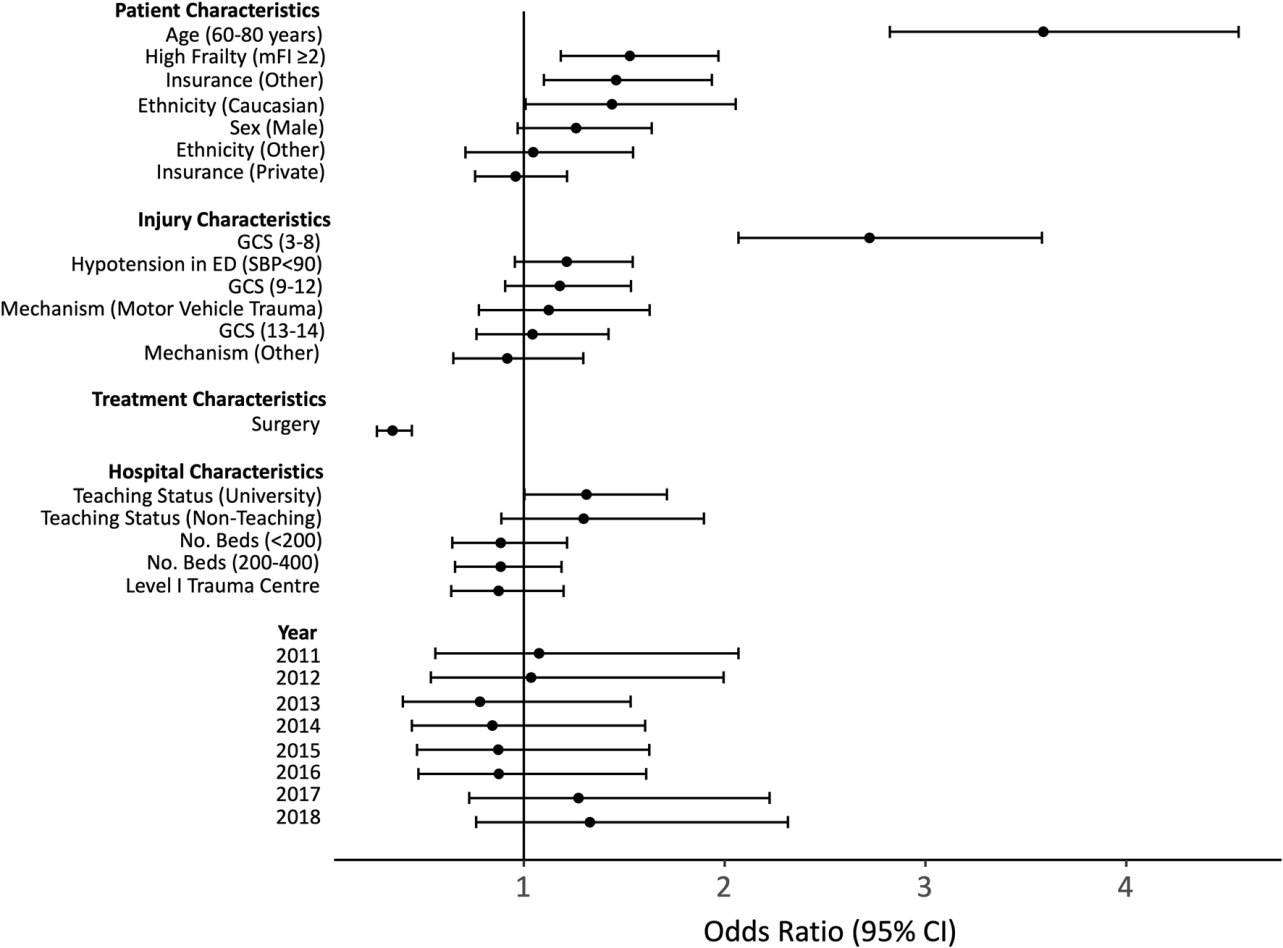
Ranked Forest plot of adjusted odds ratios and 95% confidence intervals for patient, injury, treatment, hospital, characteristics for mortality in complete cervical spinal cord injury. The depicted odds ratio for age is associated with a change from 60 to 80 years. High Frailty mFI-5 ≥ 2; Low Frailty mFI < 2. Abbreviations: mFI, Modified Frailty Index- 5; GCS, Glasgow Coma Scale; ED, emergency department; SBP, systolic blood pressure.

Within the cohort, 751 (15.87%) patients suffered an in-hospital mortality. A plot of unadjusted morality rates is given in Figure 2a. The figure demonstrates that for a given age, increased frailty results in higher crude mortality rates. As well, for a given frailty category older age results in higher crude mortality rates. Results from the full logistic regression model for mortality are summarized in Table 2 and Figure 2b. Patient characteristics significantly associated with in-hospital mortality included age (80 years vs. 60 years: OR 3.59 95% CI [2.82 4.56], *P* < 0.001); high frailty (OR 1.53 95% CI [1.19 1.97], *P* < 0.001); and Caucasian ethnicity (OR 1.46 95% CI [1.10 1.94], *P* = 0.009). Injury characteristics associated with in-hospital mortality included GCS 3–8 (OR 2.72 95% CI [2.07 3.58], *P* < 0.001). In contrast, patients who underwent surgery were associated with significantly less in-hospital mortality (OR 0.35 95% CI [0.27 0.44]). We did not find any significant effect from patient sex, year of injury, or hospital characteristics on rates of in-hospital mortality after complete cervical SCI. Figure 3 demonstrates provides a ranking of the effect size of each covariate on in-hospital mortality, stratified by covariate type. Among all covariates that were adjusted, age had the largest effect on in-hospital mortality (OR 3.59 95% CI [2.82 4.56], *P* < 0.001). Conversely, surgery was associated with the greatest reduction of in-hospital mortality (OR 0.34 95% CI [0.26 0.43]).

**Table 2:** Results from the full model testing for association of patient, injury/presentation, treatment, and hospital characteristics with in-hospital mortality for complete cervical spinal cord injury.

	OR	95% CI	*P* value
Patient characteristics			
Age (Increase from 60 to 80 y)	3.59	2.82–4.56	< 0.001
Modified frailty index-5 (mFI) category			
Low Frailty (mFI < 2)	Reference		0.001
High Frailty (mFI ≥ 2)	1.53	1.19–1.97	
Sex			
Female	Reference		0.083
Male	1.26	0.97–1.64	
Ethnicity			
African American	Reference		0.009
Caucasian	1.46	1.10–1.94	
Other	1.05	0.71–1.54	
Insurance			
Government	Reference		0.075
Private	0.96	0.76–1.22	
Other	1.44	1.01–2.05	
Injury/presentation characteristics			
Mechanism of injury			
Fall	Reference		0.149
Motor vehicle trauma	1.21	0.96–1.54	
Other	0.92	0.65–1.30	
GCS			
15	Reference		< 0.001
13–14	1.04	0.76–1.42	
9–12	1.12	0.78–1.63	
3–8	2.72	2.07–3.58	
Hypotension in ED (SBP < 90)-n (%)	1.18	0.91–1.53	0.215
Year			
2010	Reference		0.227
2011	1.08	0.56–2.07	
2012	1.04	0.54–2.00	
2013	0.78	0.40–1.53	
2014	0.84	0.44–1.60	
2015	0.87	0.47–1.62	
2016	0.88	0.48–1.61	
2017	1.27	0.73–2.22	
2018	1.33	0.76–2.31	
Treatment characteristics			
Surgery	0.35	0.27–0.44	< 0.001
Hospital characteristics			
Level I trauma center	0.89	0.66–1.19	0.417
Number of beds			
> 400	Reference		0.688
200–400	0.87	0.64–1.20	
≤ 200	0.89	0.64–1.22	
*Teaching status*			
Community	Reference		0.083
Non-teaching	1.3	0.89–1.90	
University	1.31	1.00–1.71	

OR, odds ratio; CI, confidence interval; GCS, Glasgow Coma Scale; ED, emergency department; SBP, systolic blood pressure.

### Frailty provides minimal incremental prognostic value over age

**Figure 4:**
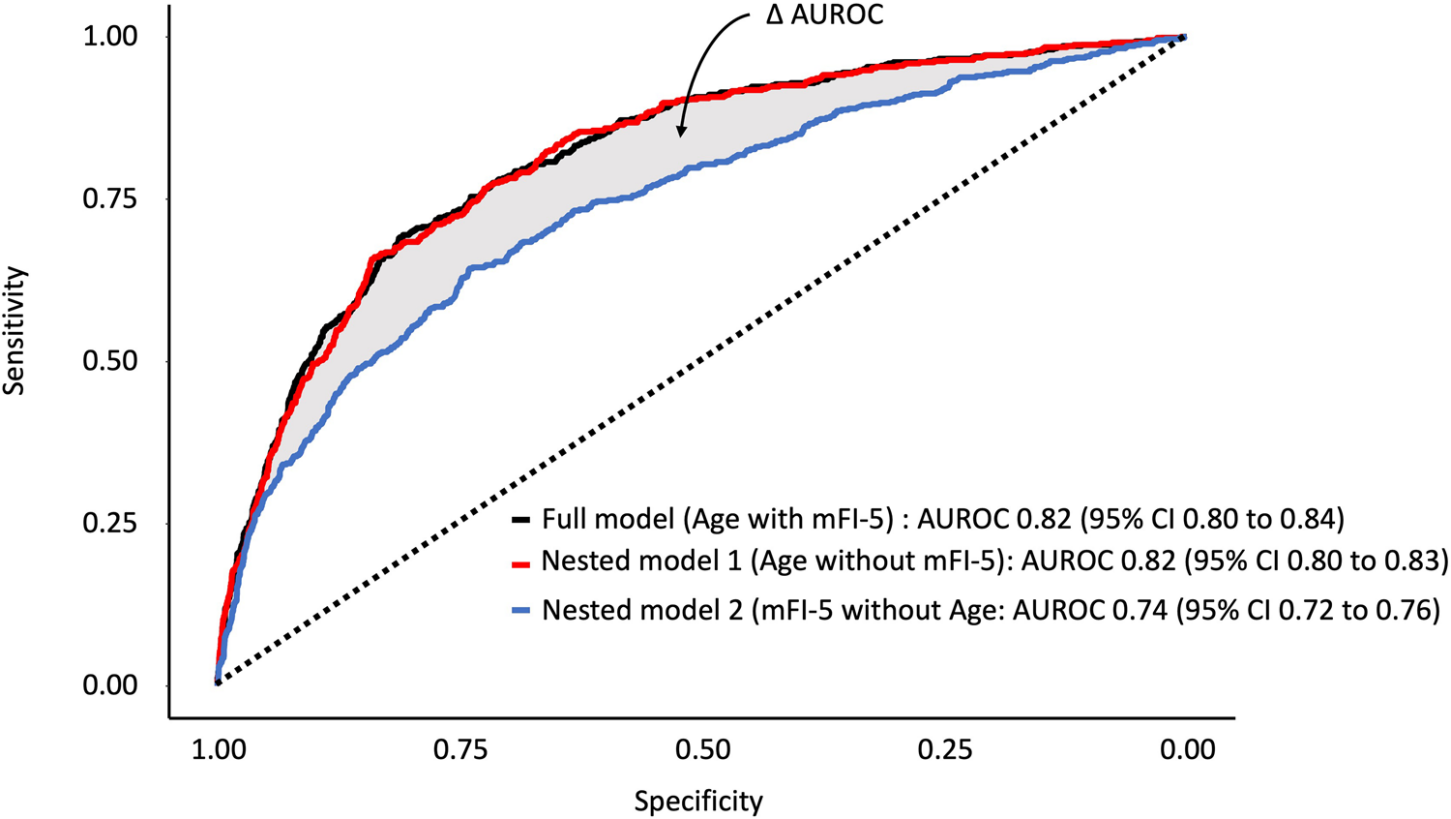
Receiver Operating Characteristic curve analysis for three regression models. A full model with all covariates was compared with nested models including either age or mFI-5 with the following 11 base covariates: sex, ethnicity, insurance type, mechanism of injury, presenting GCS, presence of shock, whether they underwent surgery, hospital ACS verification level, teaching status, hospital size, and year of injury. The full model includes age & mFI-5 with the 11 base covariates. Nest model 1 includes age along with the 11 base covariates without mFI-5. Nested model 2 incudes mFI-5 along with the 11 base covariates without age. ΔAUROC = AUROC_full model_−AUROC_nested model i_. Abbreviations: AUROC, Area under the Receiver Operating Characteristic; CI, confidence interval; mFI-5, Modified Frailty Index-5; GCS Glasgow Coma Scale; ACS American College of Surgeons.

Figure 4 and Table 3 illustrate the results of ROC analysis from three regression models comparing age and mFI-5. When comparing the estimated log-likelihood of our three models, the likelihood ratio test demonstrated a significant difference between a full model including age & mFI-5 compared to a nested model including age without mFI-5 (*P* = 0.001). We also noted a significant difference between the full model and a nested model including mFI-5 without age (*P* < 0.001). However, there was a smaller difference in the estimated log likelihood of the full model when compared to the nested model including age without mFI-5, as demonstrated by the smaller magnitude χ^2^ approximation.

**Table 3:** Assessment of incremental prognostic value from mFI-5.

	Full model (Age & mFI-5, with covariates)	Nested model 1 (Age with covariates)	Nested model 2 (mFI-5 with covariates)
Complete cohort			
Likelihood ratio test	Reference	χ^2^ = 10.54, *P* = 0.001	χ^2^ = 276.72, *P* < 0.001
ΔAUROC 95% CI	Reference	− 8.72 × 10^–4^ to 0.82, *P* = 0.199	0.06–0.09, *P* < 0.001
Somers’ D_XY_	0.61	0.61	0.45
Brier’s score	0.10	0.10	0.11
AIC	2587	2596	2858
Subgroup 1: Age ≥ 60 years			
Likelihood ratio test	Reference	χ^2^ = 4.11, *P* = 0.043	χ^2^ = 64.40, *P* < 0.001
ΔAUROC 95% CI	Reference	0.00–0.01, *P* = 0.164	− 0.06 to − 0.02, *P* < 0.001
Somers’ D_XY_	0.43	0.42	0.34
Brier’s score	0.18	0.18	0.19
AIC	1402	1404	1460
Subgroup 2: Age ≥ 60 years and Underwent Surgery			
Likelihood ratio test	Reference	χ^2^ = 5.03, *P* = 0.025	χ^2^ = 53.73, *P* < 0.001
ΔAUROC 95% CI	Reference	− 0.00–0.01, *P* = 0.357	0.03–0.09, *P* < 0.001
Somers’ D_XY_	0.34	0.33	0.20
Brier’s score	0.17	0.18	0.18
AIC	1099	1102	1147

Results are shown from 500 iteration bootstrap validation tests for regression models of in-hospital mortality in patients with complete cervical spinal cord injury. A full model with all covariates was compared with nested models including either age or mFI-5 with the following covariates: sex, ethnicity, insurance type, mechanism of injury, presenting GCS, presence of shock, whether they underwent surgery, hospital ACS verification level, teaching status, hospital size, and year of injury. ΔAUROC = AUROC_full model_−AUROC_nested model i_. Abbreviations: AIC, Akaike Information Criterion; AUROC, Area under the Receiver Operating Characteristic; CI, confidence interval; mFI-5, Modified Frailty Index-5; GCS Glasgow Coma Scale; ACS American College of Surgeons.

With respect to discrimination, we failed to find a significant difference in the AUROC between a full model with age and mFI-5 compared to a nested model including age without mFI-5 (95% CI Δ AUROC [− 8.72 × 10^-4^ 0.82], *P* = 0.199). However, a full model with age age mFI-5 demonstrated significantly superior discrimination compared to a nested model including mFI-5 without age (95% CI Δ AUROC [0.06 0.09], *P* < 0.001). Moreover, a nested model including age without mFI-5 demonstrated significantly superior discrimination compared to a nested model including mFI-5 without age (95% CI Δ AUROC [0.06 0.09], *P* < 0.001). Bootstrap validation estimates of the Somer’s D_xy_ also demonstrated superior discrimination from the full model compared to both nested models. The full model demonstrated equivalent reliability to the nested model including age without mFI-5. This was evident by the equivalent Brier’s scores computed by a bootstrap with 500 iterations. Both models demonstrated superior reliability compared to the nested model including mFI-5 without age, which had a larger Brier’s score. The full model demonstrated the best fit to the data, as it had the lowest AIC. Interestingly, the AIC of the full model was comparable to the nested model including age without mFI-5. Our findings were robust to sensitivity analysis among subgroup 1 (Age ≥ 60 years) and remained consistent in subgroup 2 (Age ≥ 60 years and underwent surgery) (Table 3).

## Discussion

In this retrospective cohort study of a large multi-center trauma database, we fit three multivariable logistic regression models for in-hospital mortality in patients with complete traumatic cervical SCI. We compared a full model including age, mFI-5, and 11 base covariates with a nested model including age and the 11 covariates without mFI-5, and a separate nested model including mFI-5 with the 11 covariates without age. The full model demonstrated that both age and high frailty independently are associated with increased in-hospital mortality in patients suffering from complete traumatic cervical SCI. Our analysis comparing the full model with the nested models demonstrates minimal incremental prognostic value derived from mFI-5 over age.

In-hospital mortality for patients suffering from traumatic cervical SCI has been estimated between 4 and 26%^1^. In our study we noted an unadjusted in-hospital mortality rate of 15.42%, consistent with what has been reported in the literature. After investigating the characteristics of patients, we noted higher rates of mortality in older patients, and patients with increased frailty as measured by mFI-5 (Figure 2). A retrospective study conducted by Blex et al. looked at specific disease predictors of in-hospital mortality in the setting of SCI^27^. Their study consisted of 321 patients from a single level 1 trauma center spanning 2011–2017. They noted an overall mortality of 6.2% within older age patients. Moreover, a higher Carlson Comorbidity Index was noted among patients who died. Our results build upon these findings, as a multi-center observational study spanning 2010–2018, including 4733 patients and calculating adjusted mortality across various age and frailty categories. In contrast to the study conducted by Blex et al., we chose to use the mFI-5 rather than the Charlson’s Comorbidity index to adjust for patient co-morbidities. The mFI-5 has been shown to be a relevant comorbidity and frailty index within the trauma population, as well as an important mortality predictor^28^.

The association of clinical frailty with poor outcomes among patients with spinal pathology has been frequently studied^29–33^. However, most of these studies pertain to degenerative spine disease, with more limited evidence in the setting of acute SCI. One retrospective multi-center cohort study conducted by Elsamadicy et al. in 2021 assessed the impact of frailty as an independent predictor of mortality in patients with cervical SCI^5^. Their study consisted of 8986 patients from 2017 that sustained cervical SCI and noted a significant effect of an mFI ≥ 2 in a logistic regression model for patient mortality (OR 1.45 95% CI [1.14 1.83]). Our study findings are consistent with this result, with a similar effect size for mFI ≥ 2 (OR 1.53 [1.19 1.97]). However, in addition to investigating frailty as a predictor of mortality, we assess its incremental prognostic value relative to age. We show that in a base mortality model, adding patient frailty as a covariate produces a smaller AUROC when compared to the addition of age (Table 3). Moreover, once age is included in a regression model, the addition of frailty does not seem to provide further improvement to the AUROC of the model. This result was robust to sensitivity analyses within older age and surgical patient subgroups. These findings suggest that among patients with complete SCI, age provides superior discrimination than frailty for in-hospital mortality prediction. This contrasts with findings published by Wilson et al. that suggests frailty provides superior prediction of post-operative mortality and complications relative to age among patients undergoing surgery for degenerative cervical myelopathy. This difference may be due to differences in the patient populations that suffer from each of these conditions. The mean age of patients in our cohort was 49.05, with most patients presenting with low frailty represented by an mFI-5 less than 2. This is likely different from the population of patients that suffer from degenerative cervical myelopathy, which could impact the relationship between these variables and mortality. Further, in our study we assessed all-cause in-hospital mortality, whereas Wilson et al. looked at 30-day mortality. Assessing mortality after a longer follow-up period than what was used in our study may alter findings. This represents a limitation of the TQIP database, where all data is limited to the in-hospital trauma admission. Lastly, a differential selection bias for surgery among patients with degenerative spine disease and complete traumatic cervical SCI may also account for differences in our findings from what has been found in patients with cervical myelopathy. Certainly, surgery for degenerative spine disease can often be done on an elective basis, whereas surgery for traumatic cervical SCI is done urgently. This difference, along with differences in the selection bias for surgery relative to patient frailty may result in changes in the relationship between frailty and in-hospital mortality.

Age has been shown to be a predictor of mortality in the setting of acute traumatic SCI^34,35^. A long-term survival study conducted by Frankel et al. investigated mortality rates of 3,179 patients who suffered acute SCI^34^. Their study spanned 50 years and provided long term survival data. Using Cox proportional hazards regression, they noted an increased risk of mortality with a higher age at the time of injury (risk ratio 1.07 95% CI [1.07 1.08]). A separate retrospective cohort study by Inglis et al. utilizing the Rick Hansen Spinal Cord Injury Registry used logistic regression to compute predicted in-hospital mortality among patients with traumatic SCI who underwent surgery^35^. They found older age (≥ 77 years) to be a significant predictor (*P* < 0.001) of in-hospital mortality among these patients (OR 6.76 95% CI [3.04 15.05]). Our results are consistent with these findings, whereby we noted that older age was a significant predictor (*P* < 0.001) of in-hospital mortality (OR 3.59 95% CI [2.82 4.56], *P* < 0.001). In addition to contributing more evidence to the impact of age on mortality among patients with SCI, we found that age and mFI-5 are each associated with increased in-hospital mortality after covariate adjustment.

A strength of this study is the use high-quality audited data across various trauma centers within North America. Notably, we did not find a significant effect from ACS verification level, hospital size (number of beds), or teaching status in our regression model (Table 3). This finding improves the applicability of these results to various institutions. Moreover, the large size sample size of nearly 5000 patients enabled adjustment of predicted in-hospital mortality to numerous patient and trauma characteristics. However, there are some notable limitations to our findings. Given the observational and retrospective study design, we cannot conclude any causal effects between regression model covariates and in-hospital mortality. Second, our study design meant that our assessment of mortality was limited to the in-hospital trauma admission. This was largely due to the data available within TQIP. There may be a larger proportion of patients within each age category that die within a short interval after hospital discharge or transfer. This highlights the need for short term follow-up studies in this patient population to determine interval mortality rates for each patient subgroup. Finally, our measurement of frailty was limited to the mFI-5. Although this index has been shown to be relevant in the context of mortality in trauma, it does not completely capture every dimension of reduced physiologic reserve encompassed in a clinically frail patient. Other measures of clinical frailty exist, such as the 9-point Clinical Frailty Scale, the 11-point Modified Frailty Index, the Fried Phenotype model, and the accumulating deficit model of frailty^14,36,37^. Our findings may be limited to the mFI-5, such that other models of frailty exhibit incremental prognostic value for prediction of in-hospital mortality. However, capturing the level of patient data required for measurement of alternative frailty indices remains a challenge in the setting of trauma, where patients are often unidentified^38^.

Increasing age and frailty are associated with increased in-hospital mortality among patients suffering complete cervical SCI. In the current study we have shown that when comparing these two variables in a mortality regression model, mFI-5 yields minimal incremental prognostic value. The results of these studies will hopefully serve to assist in counselling of SCI patients and their families, and in the future may help direct the establishment of improved treatment and triaging protocols for spinal trauma.

## Supplementary Information

Supplementary Information.

## Data Availability

The data that support the findings of this study are available from American College of Surgeons (ACS) Trauma Quality Improvement Program (TQIP) but restrictions apply to the availability of these data, which were used under license for the current study. Data from this study is owned by the ACS and it is publicly available, but a request has to be made to the ACS. The corresponding author of this paper can be contacted for guidance in requesting access to this data.
