# Supplementary material for: Assessment of the incremental prognostic value from the modified frailty index-5 in complete traumatic cervical spinal cord injury
Source: Sci Rep. 2023 May 10;13:7578. doi: 10.1038/s41598-023-34708-5 (PMC10172291; doi:10.1038/s41598-023-34708-5)
Supplement: Supplementary file 1 — Supplementary Information. [file 41598_2023_34708_MOESM1_ESM.pdf]

Assessment of the Incremental Prognostic Value from the Modified Frailty Index-5 in Complete Traumatic Cervical Spinal Cord Injury

Husain Shakil, MD, MSc[1-4], Blessing N.R. Jaja, MD, PhD[2], Peng F. Zhang, BSc[2], Rachael H. Jaffe[2,4], Armaan K. Malhotra, MD[1-4], Erin M. Harrington, BMSc[2], Duminda N. Wijeysundera, MD, PhD[2,4,5,6] Jefferson R. Wilson, MD, PhD[1-4], Christopher D. Witiw, MD, MSc[1-4, *]

[1]University of Toronto, Department of Surgery, Division of Neurosurgery, Toronto, M5T1P5, Canada
[2]St. Michael's Hospital, Li Ka Shing Knowledge Institute, Toronto, M5B1T8, Canada
[3]St. Michael's Hospital, Department of Surgery, Division of Neurosurgery, Toronto, M5B1W8, Canada
[4]University of Toronto, Institute of Health Policy Management and Evaluation, Toronto, M5T1P8, Canada
[5]St. Michael's Hospital, Department of Anesthesia, Toronto, M5B1W8, Canada
[6]University of Toronto, Department of Anesthesiology and Pain Medicine, Toronto, M5T1P8, Canada
[*]Christopher.Witiw@unityhealth.to

Acute Injury Severity codes for Complete Cervical Spinal Cord Syndrome [a]	
	640220.5, 640221.5, 640222.5, 640224.5, 640226.5, 640228.5, 640229.6, 640230.6, 640232.6, 640234.6, 640236.6; 640260.5, 640261.5, 640262.5, 640264.5, 640266.5, 640268.5, 640269.6, 640270.6, 640272.6, 640274.6, 640276.6

[a] Quadriplegia or paraplegia with no sensation

Supplementary Table S1. Acute Injury Severity codes used to identify complete cervical spinal cord injury patients within the Trauma Quality Improvement Program.

International Classification of Diseases 9 and 10 Procedural Classification System Codes		
Procedure	ICD-9-PCS Codes	ICD-10-PCS Codes
Decompression	3.01, 3.02, 3.09, 3.53	0RN0, 0RN1, 0RN3, 0RN4, 0RN5, 0RT3, 0RT4, 0RT5, 0RTB, 0RP0, 0RP1, 0RP3, 0RP4, 0RP5, 0RB0, 0RB1, 0RB3, 0RB4, 0RB5, 0RW0, 0RW1, 0RW3, 0RW4, 0RW5, 0RW9, 0RQ0, 0RQ1, 0RQ3, 0RQ4, 0RQ5, 00NW
Fusion	81.00, 81.01, 81.02, 81.03, 81.30, 81.31, 81.32, 81.33 81.62, 81.63, 81.64	0RG0, 0RG1, 0RG2, 0RG4, 0RH0, 0RH1, 0RH3, 0RH4, 0RH5, XRG0, XRG1, XRG2, XRG4

Abbreviations: ICD, International Classification of Diseases; PCS, Procedural Classification System.

Supplementary Table S2. International Classification of Diseases 9 and 10 Procedural Classification System Codes used to identify patients who underwent surgery related to their complete cervical spinal cord injury.

Predictor	P-Value
Age – Composite Predictor	<0.001
Age – Non-linear terms	0.006
mFI-5 Category	0.001
Sex	0.083
Ethnicity	0.008
Insurance	0.075
Mechanism of Injury	0.149
GCS	<0.001
Hypotension in ED (SBP < 90)	0.215
Year	0.227
Surgery	<0.001
Level I trauma center	0.417
Number of beds	0.688
Teaching status	0.083

Abbreviations: mFI-5, Modified frailty index 5; GCS, Glasgow Coma Scale; ED, emergency department; SBP, systolic blood pressure.

Supplementary Table S3. Results from the analysis of variance for predictors within a multivariable logistic regression model for in-hospital mortality in patients with traumatic cervical spinal cord injury, with age modelled as a non-linear predictor for in-hospital mortality.

	χ^2	*P Value*
+/- Non-linear age terms	10.08	0.006
+/- Age-mFI-5 category interaction terms	5.21	0.157

Abbreviations: mFI-5, Modified frailty index 5.

Supplementary Table S4. Results from likelihood ratio test comparing models of various complexities. Models with and without non-linear terms for age are compared and demonstrate a significant difference in their log-likelihood based on the likelihood ratio test. Models with and without an interaction term for age and modified frailty index-5 (mFI-5) category are compared and fail to demonstrate a significant difference in their log-likelihood based on the likelihood ratio test.

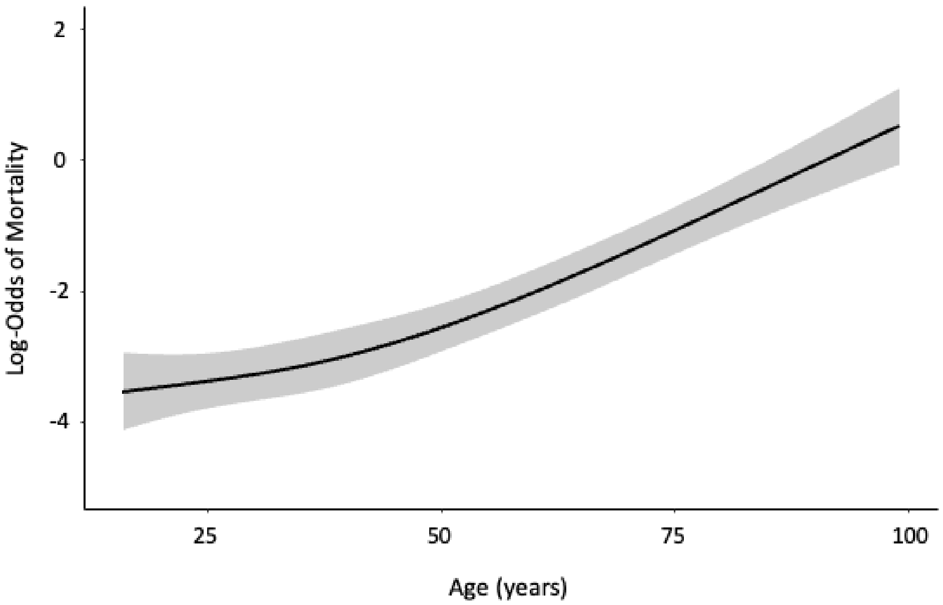

Supplementary Figure S5. Results from the full model demonstrating a non-linear relationship between the log-odds of in-hospital mortality and age. Plots are adjusted to: mFI-5 < 2, male sex, caucasian ethnicity, private insurance, absence of hypotension, fall as mechanism of injury, GCS 15, hospital bed size ≤ 200, level 1 American College of Surgeon verification level hospitals, university hospitals, injury year 2017, and patients undergoing surgery.

**Predictor**	**P-Value**
Age – Composite Predictor	<0.001
Age – All Interactions	0.209
Age – Non-linear terms	0.005
mFI-5 Category – Composite Predictor	0.002
mFI-5 Category – All Interactions	0.209
Age- mFI-5 Category Interaction Term – Composite Predictor	0.209
Age- mFI-5 Category Interaction Non-linear Terms	0.153
Sex	0.075
Ethnicity	0.009
Insurance	0.065
Mechanism of Injury	0.185
GCS	<0.001
Hypotension in ED (SBP < 90)	0.231
Year	0.188
Surgery	<0.001
Level I trauma center	0.385
Number of beds	0.697
Teaching status	0.074

Abbreviations: mFI-5, Modified frailty index 5; GCS, Glasgow Coma Scale; ED, emergency department; SBP, systolic blood pressure.

Supplementary Table S6. Results from the analysis of variance for predictors within a multivariable logistic regression model for in-hospital mortality in patients with traumatic cervical spinal cord injury, with age modelled as a non-linear predictor for in-hospital mortality and including interaction terms between age and mFI-5.
